# Climate-resilient *Durum wheat* selection using explainable machine learning and multi-environment trials

**DOI:** 10.3389/fpls.2026.1796352

**Published:** 2026-05-26

**Authors:** Nadia Nawel Azizi, Louiza Smichette, Nesrine Hacini, Mohamed Nejib El Melki, Jameel M. Al-Khayri, Bader Alsubaie, Othman Al-Dossary, Mohammed I. Aldaej

**Affiliations:** 1 Laboratory of Functional and Evolutionary Ecology Research, Faculty of Nature and Life Sciences, Chadli Bendjedid University of El Tarf, El-Tarf, Algeria; 2 Higher School of Engineers of Medjez El Bab, Department of Mechanical and Agro-Industrial Engineering, University of Jendouba, Jendouba, Tunisia; 3 Department of Agricultural Biotechnology, College of Agriculture and Food Sciences, King Faisal University, Al-Ahsa, Saudi Arabia

**Keywords:** climate change, durum wheat, genotype × environment interaction, machine learning, SHAP analysis, yield stability

## Abstract

**Introduction:**

Climate change poses a growing threat to durum wheat production in Mediterranean semi-arid regions, mainly through rising temperatures, frequent water shortages, and increased genotype × environment interactions.

**Methods:**

Over five consecutive growing seasons (2021–2025), ten durum wheat genotypes were evaluated across three rainfed environments in Algeria: Oued Smar, Sétif, and El Khroub. Agronomic performance, yield stability, and grain quality were assessed using ANOVA, AMMI, ASI, GGE biplots, drought tolerance indices (STI, DSI, and YSI), and supervised machine learning models coupled with SHAP-based interpretation.

**Results:**

Environment, genotype, and genotype × environment interaction had highly significant effects on grain yield. Yield differences exceeded 50% between sites. Waha and Skh/hau.heca-1 were identified as broadly adapted and stable genotypes, whereas Ammar-8, Da-6blak, and Lahaucan showed high yield potential but stronger environmental sensitivity. SHAP analysis indicated that precipitation and water deficit were the main drivers of yield variability, together explaining 50% of model importance, followed by grain number and soil plant-available water capacity.

**Discussion:**

The integration of stability analysis, drought tolerance indices, and explainable machine learning provides a robust framework for identifying climateresilient durum wheat genotypes and guiding targeted varietal deployment under Mediterranean rainfed conditions.

## 1 Introduction

The Mediterranean Basin is now considered a critical “hotspot” for global climate change, facing unprecedented risks to agricultural sustainability and food security Zandalinas et al. (2021). According to climate projections, temperatures are expected to rise by 2–4 °C, accompanied by a 20–30% reduction in precipitation by the year 2050. At the same time, extreme weather events, such as severe droughts, are expected to intensify in the future, as well as the worsening of climate change symptoms IPCC (2021); Zittis et al. (2016). These shifts are estimated to cause a significant drop in cereal production worldwide; some forecasters predict a 6% decrease in the yield of wheat per degree Celsius of the increase in global temperature Asseng et al. (2015); Zhao et al. (2017). These ecological changes present significant structural pressure to rainfed agricultural systems that form the backbone of agriculture in North Africa. The importance of cereal cultivation cannot be ignored because it is directly linked to the amount of precipitation. Therefore, to maintain stability in the region and food security, it is important to ensure that these basic crops are resilient.

In the Algerian case, this weakness is worsened by the extreme variability in climatic conditions, thus destabilizing agricultural production and undermining yearly output. This climatic change is particularly worrying because Algeria has some of the highest levels of per-capita consumption of wheat globally, often exceeding 200 kg/year/capita. The subsequent structural food dependence and rapid population growth have put an ongoing strain on national stocks and increased the cost of imports when national production is insufficient in the present geopolitical environment of tensions between the major cereal producers Russia and Ukraine.

*Triticum durum* Desf. is a central factor in the food security of Algeria Mekhlouf et al. (2022); El Hassouni et al. (2018). However, the yield does not usually surpass 2 t ha^−1^, which is significantly lower than the known genetic potential of 5t ha^−1^, in spite of the fact that it is mostly grown in rainfed semi-arid areas Rahimi et al. (2014).

The increasing disparity between the domestic demand and national production of *Durum wheat* reflects the need to stabilize productivity and enhance our understanding of the genotype-environment (G×E) relationships in the context of recurrent climatic events Senoussi et al. (2020). Statistical models based on a linear or multiplicative form, including ANOVA, AMMI models, or Finlay-Wilkinson regressions, are traditionally used to evaluate varietal stability through multi-environmental trials (MET) Gauch and Zobel (1988); Olivoto et al. (2019). However, such traditional methods do not always reflect the nonlinear relationships between the genetic background and environmental variation, which are the result of the synergy between the genetic background and environmental variations Malosetti et al. (2013). Machine learning is a suitable alternative for analysis that can model multidimensional relationships and the primary predictors of genetic performance in climate change scenarios Montesinos-L’opez et al. (2021); Lundberg and Lee (2017). The combination of these smart approaches with stress tolerance indices (e.g., STI, DSI, and YSI) and GGE biplots can help to conduct an assessment of genotypic performance and stability in changing climatic conditions in a more detailed manner Yan (2011). Algeria has limited scientific research in terms of incorporating traditional statistical techniques, which are in line with current advanced techniques for analyzing data. This inadequacy impairs climate change mitigation measures and the parameters used to select the existing variety Mohammadi et al. (2022).

Furthermore, multi-environmental trials (METs) still do not sufficiently take into account the simultaneous characterization of grain yield and end-use quality attributes, such as protein content, wet gluten content, Zeleny sedimentation index, and specific weight, under contrasting water deficit conditions, despite their direct relevance to semolina milling efficiency, pasta cooking quality, and commercial grading standards in the durum wheat processing industry López-Bellido et al. (1998); De Santis et al. (2021).

This study aimed to evaluate ten durum wheat genotypes, including improved cultivars and local varieties, in three different rainfed contexts in Algeria, over five growing seasons (2021–2025). The objectives were: (1) to use statistical and stability analyses (ANOVA, AMMI, ASI, and GGE biplots) to assess the effects of genotype, environment, and their interaction on grain yield and its components; (2) to use supervised machine learning algorithms (Random Forest, SVM, XGBoost) with SHAP for model interpretability to identify the main environmental and agronomic factors of yield variability; and (3) to create a multi-criteria classification system for genotypes to provide targeted varietal recommendations. In order to study the yield-quality trade-offs under progressive water deficit conditions, grain quality parameters such as protein content, wet gluten content, Zeleny sedimentation index, and specific weight were examined as secondary variables. voir aussi cette partie.

## 2 Materials and methods

### 2.1 Experimental sites, climatic and soil characteristics

**Figure 1:**
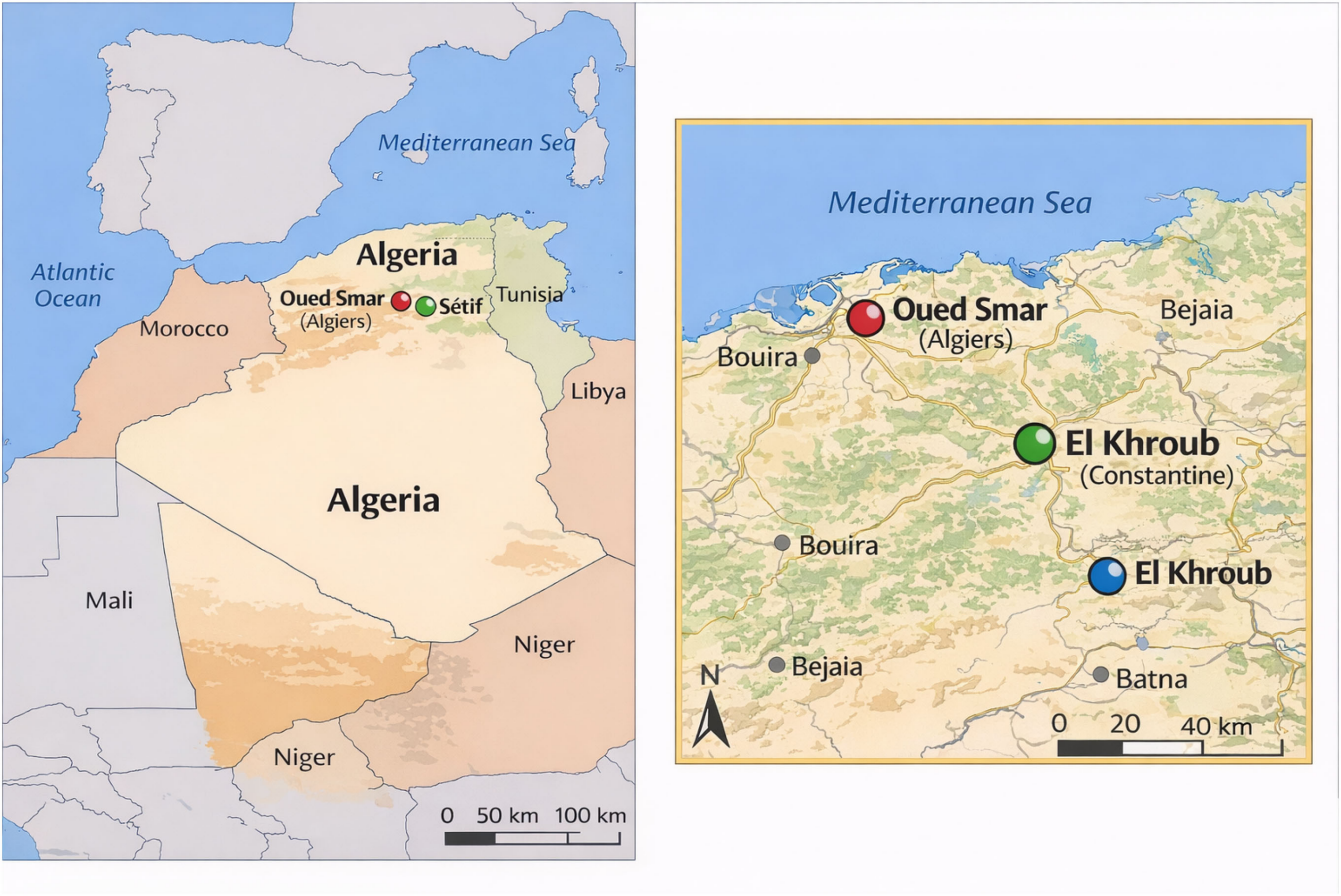
Geographical locations of the three experimental sites used in the MET, multi-environment trial of *durum wheat* in Algeria (2021–2025). E1: Oued Smar (36.72°N, 3.14°E, alt. 24m); E2: Sétif (36.19°N, 5.41°E, alt. 1023m); E3: El Khroub (36.26°N, 6.69°E, alt. 570m). CWD: cumulative water deficit; SWC: soil water content.

The trials of cereal crops were conducted over five consecutive growing seasons (2021–2025), with four replicates per site per season, at three agroclimatic sites that represent diverse soil and climate conditions of cereal production in Algeria: Oued Smar (E1), Sétif (E2), and El Khroub (E3) (**Figure 1**). The selected sites were chosen to represent the least and most water-stressed conditions. The Algerian National Meteorological Office’s monthly bulletins provided the climatic data (Office National de Met´ eorologie´ (ONM), 2025). Each experimental site’s soil water content (SWC) was measured directly using the gravimetric method, and the cumulative water deficit (CWD) was determined using the method of Doorenbos and Kassam (1979). El Khroub (E3) had the lowest water stress (CWD = 150 mm), despite receiving the least amount of rainfall (∼350 mm), because of its favorable soil water retention capacity. Sétif (E2) experienced moderate water stress (CWD = 380 mm, rainfall ∼400 mm). Oued Smar (E1), situated in the Mitidja Plain, receives approximately 600 mm of annual rainfall but has the highest cumulative water deficit (CWD = 560 mm), indicating the most severe water stress among the three sites.

Because of its superior soil water retention capacity, El Khroub (E3) has the lowest cumulative water deficit (CWD = 150 mm) among the three sites, although receiving the least annual rainfall (∼350 mm). In comparison, Sétif (E2) shows an intermediate scenario (CWD = 380 mm, rainfall ∼400 mm), but Oued Smar (E1) records the most severe water stress (CWD = 560 mm) despite getting the highest rainfall (∼600 mm). These disparate circumstances demonstrate that the water stress gradient across the three experimental sites is determined by soil characteristics and evapotranspiration demand rather than only rainfall (**Table 1**).

**Table 1:** Main physico-chemical characteristics of the soils at the three experimental sites (0–30 cm depth).

Site	Texture	pH	EC (dS m^−1^)	OM (%)	Total N (%)	Available P (mg kg^−1^)	CEC (cmol kg^−1^)
Oued Smar (E1)	Clay-loam	7.2	0.4	2.1	0.16	28.5	26.4
Sétif (E2)	Clay (Vertisol)	7.9	0.7	1.4	0.11	18.3	23.1
El Khroub (E3)	Loamy-calcareous	8.2	0.9	1.1	0.08	12.6	19.5

EC, electrical conductivity; OM, organic matter; N, total nitrogen; P, available phosphorus; CEC, cation exchange capacity.

The geographical coordinates of Oued Smar are 36.72° N, 3.14° E, located at an altitude of 24m. The geographical coordinates of Sétif are 36.19° N and 5.41° E, located at an altitude of 1023m. Finally, the geographical coordinates for El Khroub are 36.26° N, 6.69° E, located at an altitude of 570m.

For each replicate (2021–2025) at each site, throughout the plant growth period from sowing to harvest (October to June), meteorological conditions were recorded daily using automatic weather stations. The recorded variables included the air temperature (minimum and maximum), relative humidity, wind speed, and incident solar radiation. Reference evapotranspiration (ET_0_) was calculated using the FAO-56 PenmanMonteith reference equation (Allen et al., 1998). Crop water deficit (CWD) is defined as the cumulative ET_0_ minus the actual rainfall, which was estimated using the USDA SCS curve number method (USDA, 1985) as follows: The soil particle size distribution in a 30cm deep layer was determined using the Bouyoucos hydrometer method. According to the analysis of the particle size, three different types of soils were identified, including silty clay at Oued Smar, heavy vertisol at Sétif, and silty calcareous in El Khroub. The soil water retention capacity (SWC) was estimated using pedotransfer functions by integrating textural data (clay, silt, sand) and average bulk density measured at various points within each site. The calculation was performed for the estimated effective rooting depth of durum wheat at each site. The results showed values of 190mm in Oued Smar (E1), 140mm in Sétif (E2), and 115mm in El Khroub (E3). Other physicochemical properties, such as pH and organic matter content, are listed in **Table 1**.

### 2.2 Plant material and experimental setup

Using a Randomized Complete Block Design (RCBD) with four replicates per site, ten durum wheat genotypes — six improved cultivars and four landraces (**Table 2**) — were assessed throughout five successive growing seasons (2021–2025). Each experimental unit consisted of a 7.2 m^2^ plot (6 × 1.2m) with six rows spaced 20cm apart. The seeding rate was 140kg ha^−1^, equivalent to 350 viable seeds m^−2^. In order to prevent edge effects, all measurements were obtained from the four inner rows of each plot, and the border rows on either side were not included in the yield calculation or sampling. A plot combine harvester was used to harvest the net plot area in order to calculate the grain yield (q ha^−1^). All three sites were sown in November (November 10–20 in El Khroub, November 14–24 in Sétif, and November 18–28 in Oued Smar), and harvesting occurred between June 15 and June 25, depending on each site’s physiological maturity. Standard agronomic procedures were followed in crop management: 100kg ha^−1^ of diammonium phosphate (DAP, 18-46-0) was applied at sowing, and 60kg N ha^−1^ of nitrogen fertilizer was applied during the tillering stage (Zadoks GS 21–23). Throughout each growing cycle, weeds were manually controlled, and no fungicide, pesticide, or herbicide treatments were applied.

**Table 2:** *Durum wheat* genotypes used in the study with their origin and status.

Genotype	Type	Origin/breeding program	Reference
Waha	Landrace	Algeria	Boufenar-Zaghouane and Zaghouane (2000)
GTA dur	Improved	ICARDA/CIMMYT	ICARDA (2020)
Skh/hau.heca-1	Improved	ICARDA	Nachit and Elouafi (2004)
Ammar-8	Improved	ICARDA	Nazco et al. (2012)
MSbl_1/ourmal	Landrace	Mediterranean/Algeria	Moragues et al. (2006)
Azeghar1/6Zan-1/5	Improved	CIMMYT	Singh et al. (2011)
Villemur/3/lahn//gs	Landrace	Mediterranean	Royo et al. (2007)
Gsbl/d68-1-93-a-a1	Improved	ICARDA	Pfeiffer et al. (2000)
Lahaucan	Landrace	Algeria	Hacini et al. (2022)
Da-6blak avns/b/ber	Improved	CIMMYT/ICARDA	Ammar et al. (2004)

### 2.3 Phenological and agronomic measurements

At harvest, plant height (cm) was measured as the mean of ten randomly selected plants, and ear density (ears m^−2^) was determined by counting ears within a known area. The number of grains per ear (NGE) was averaged from ten randomly selected ears, and the thousand-grain weight (TGW, g) was determined by weighing two batches of 500 grains. Grain number per unit area (N.GRS m^−2^) was then calculated as the product of ear density and the number of grains per ear. Finally, the grain yield (kg ha^−1^) was determined for the harvested area of each experimental plot. Grain quality characteristics were analyzed using composite grain samples per plot. These included dry-base grain moisture content (%), measured using a portable moisture meter; protein content (%), determined using the Kjeldahl method (Kjeldahl, 1883); specific gravity (kg hL^−1^), measured using a standard chondrometer (AACC International, 2010); wet gluten content (%), determined using a Glutomatic 2200 system according to ISO 21415-2 (ISO, 2015); and Zeleny sedimentation (mL), measured according to ISO 5529 (ISO, 2007). Phenotypic plasticity was calculated by comparing the mean values of each genotype in different environments. The percentage variation in each characteristic between the environments (Δ*T* (%)) was calculated using Equation 1.

(1) $$\text{Δ}T(\%)=\frac{T_{Env2}-T_{Env1}}{T_{Env1}}\times 100$$

where $T_{\text{Env}1}$ and $T_{\text{Env}2}$ represent the average values of the characteristic in the two environments being compared (e.g., Oued Smar vs. Stif, Oued Smar vs. El Khroub). For this analysis, Oued Smar (E1) served as the reference environment ( $T_{\text{Env}1}$) for comparison with Sétif (E2) and El Khroub (E3) as ( $T_{\text{Env}2}$).

#### 2.3.1 Drought tolerance indices

Yields under water stress conditions (*Y_s_*) and potential conditions (*Y_p_*) were used to analyze the drought tolerance. Three indices were considered for the drought tolerance analysis: the Stress Tolerance Index (STI), Drought Sensitivity Index (DSI), and Yield Stability Index (YSI). The Khroub region (E3) was considered a site with potential yield (*Y_p_*) because of its low cumulative water deficit (150 mm) compared to other sites (380–560 mm). Considering Khroub as a “potential” site is common practice in rainfed systems when a fully irrigated control is unavailable.

The drought sensitivity index (DSI) (Fischer and Maurer, 1978) measures the yield reduction under stress conditions relative to the potential yield, which is normalized by the average stress intensity of all genotypes. A DSI *<* 1 indicates lower-than-average yield reduction (higher drought tolerance). The drought sensitivity index was calculated using Equation 2:

(2) $$\text{DSI}=\frac{1-\frac{Y_{a}}{Y_{p}}}{1-\frac{Y_{s}}{Y_{p}}}$$

where *Y_a_*is the actual yield of a specific genotype under stress conditions, *Y_p_*is the mean potential yield of all genotypes under non-stressed conditions, and *Y_s_*is the mean yield of all genotypes under stress conditions.

To identify genotypes that perform well under both stress and potential conditions, the Stress Tolerance Index (STI) (Fischer and Maurer, 1978) was employed in this study. The STI combines performance under stress conditions with potential performance. An STI value closer to or greater than 1 indicates good performance under both stress and favorable conditions. The stress tolerance index was calculated using Equation 3:

(3) $$\text{STI}=\frac{Y_{s}\times Y_{p}}{{\left(\bar{Y_{p}}\right)}^{2}}$$

Genotypes with DSI *<* 1 and STI *>* 1 were considered drought tolerant, indicating their ability to maintain productivity under reduced water availability.

Finally, yield stability was quantified using the yield stability index (YSI) Bouslama and Schapaugh (1984) (Equation 4). This is the ratio between performance under stress and potential performance. Higher YSI values indicate better stability in response to stress.

(4) $$\text{YSI}=\frac{Y_{s}}{Y_{p}}$$

Genotypes with DSI *<* 1 and STI *>* 1 were considered drought tolerant, indicating their ability to maintain productivity in environments with reduced stress susceptibility.

#### 2.3.2 Modelling genotype by environment interaction and stability

To analyze genotype-environment interactions, assess genotype stability in response to the climate gradient, and develop adaptation strategies, three statistical models were implemented: Principal Component Analysis (PCA) coupled with Analysis of Variance (ANOVA) and Additive Main Effects and Multiplicative Interaction (AMMI) Gauch and Zobel (1988). The combination of these three models allows us to decompose phenotypic variation into additive components for the main effects and a multiplicative component for the genotype × environment interaction (Equation 5).

(5) $$Y_{ij}=\mu +G_{i}+E_{j}+\sum_{k=1}^{p}\lambda_{k}\alpha_{ik}\gamma_{jk}+\epsilon_{ij}$$

where *Y_ij_*is the observed performance of genotype *i* in environment *j*, *µ* is the overall mean, *G_i_*is the effect of genotype *i*, *E_j_*is the effect of environment *j*, *λ_k_*is the singular value of the *k*-th interaction principal component axis (IPCA), *α_ik_*and *γ_jk_*are the genotype and environment eigenvectors for axis *k*, respectively, and *ϵ_ij_*is the residual error term. The term $\sum_{k=1}^{p}\lambda_{k}\alpha_{ik}\gamma_{jk}$ represents the G×E interaction through IPCA decomposition. AMMI biplots were constructed to represent these interactions. Genotypes far from the origin demonstrated strong site-specific adaptation, whereas genotypes located near the biplot origin exhibited minimal site-specific adaptation, indicating stable performance.

#### 2.3.3 Environmental characterization and site similarity

To determine the representative traits of the three sites that met environmental constraints, an analysis of trait importance (Equation 6) was performed.

(6) $${\text{Importance}}_{t}=\frac{\sigma_{Site,\;t}^{2}}{\sigma_{Total,\;t}^{2}}\times 100$$

where $\sigma_{\text{Site},t}^{2}$ is the variance due to site effects for trait *t*, and $\sigma_{Total,\;t}^{2}$ is the total trait variance, respectively. To determine the degree of environmental similarity and identify the presence of G×E “crossover” type interactions, Pearson’s correlation coefficients (*r_jk_*) were calculated for all pairs of sites for each performance indicator using Equation 7. Genotypic means were used to calculate the correlation coefficients between the two sites studied.

(7) $$r_{jk}=\frac{\sum_{i=1}^{m}(Y_{ij}-{\bar{Y}}_{\cdot j})(Y_{ik}-{\bar{Y}}_{\cdot k})}{\sqrt{\sum_{i=1}^{m}{\left(Y_{ij}-{\bar{Y}}_{\cdot j}\right)}^{2}\sum_{i=1}^{m}{\left(Y_{ik}-{\bar{Y}}_{\cdot k}\right)}^{2}}}$$

where *m* is the number of genotypes, *Y_ij_*and *Y_ik_*are the performance indicators of genotype *i* at sites *j* and *k*, respectively, and ${\bar{Y}}_{\cdot j}$ and ${\bar{Y}}_{\cdot k}$are the site means, respectively. High positive correlation coefficient values (*r >* 0.7) indicate that the ranking of genotypes was conserved across environments.

### 2.4 Machine learning models and feature importance analysis

To quantify the marginal contribution of each environmental, agronomic, and genotypic variable to the prediction of cereal yield at the three experimental sites and identify the dominant factors in different rainfall environments. Three supervised machine learning algorithms were used: Random Forest (RF), Support Vector Machine (SVM), and Extreme Gradient Boosting (XGBoost). The five-year averages (2021–2025) of environmental variables (cumulative rainfall, crop water deficit, and plant-available water capacity), agronomic traits (number of grains per m^2^ and weight of one thousand grains), and genotypic descriptors (genotype identity and genotype type) were defined as inputs for the different models. The most efficient was coupled with SHapley’s additive explanations (SHAP) to select the most appropriate combinations to meet the climate challenge. The coefficient of determination (R^2^) and root mean square error (RMSE) were used to evaluate the performance of the three models. The additive explanations of SHapley (SHAP) were applied to the machine learning model.

### 2.5 Genotype classification

A multi-criteria classification system based on the integration of AMMI analyses, drought tolerance indices, Finlay-Wilkinson stability, GGE stability performance, site correlations, and trait importance was used. The classification criteria for each of the four adaptation types are summarized in **Table 3**.

**Table 3:** Environmental stability and adaptation classification based on AMMI and regression parameters.

Type	Adaptation	|IPCA|	b_i_	S2_i_	Performance recommended use	References
I	Wide and stable	*<* 0.5	0.9–1.1	Low	High and stable All environments	Eberhart and Russell (1966); Finlay and Wilkinson (1963)
II	Favorable environments	≥ 0.5	*>* 1.1	Moderate to high	High in favorable conditions Intensive systems	Yan et al. (2000); Gauch (2006)
III	Drought-prone environments	Variable	*<* 0.9	Low to moderate	Stable under water stress Marginal zones	Ceccarelli (1989); Blum (2011)
IV	Poorly adapted	High	Variable	Very high	Low and unstable Not recommended	Lin and Binns (1988)

## 3 Results

### 3.1 Effect of environmental conditions and genotypic performance

**Figure 2:**
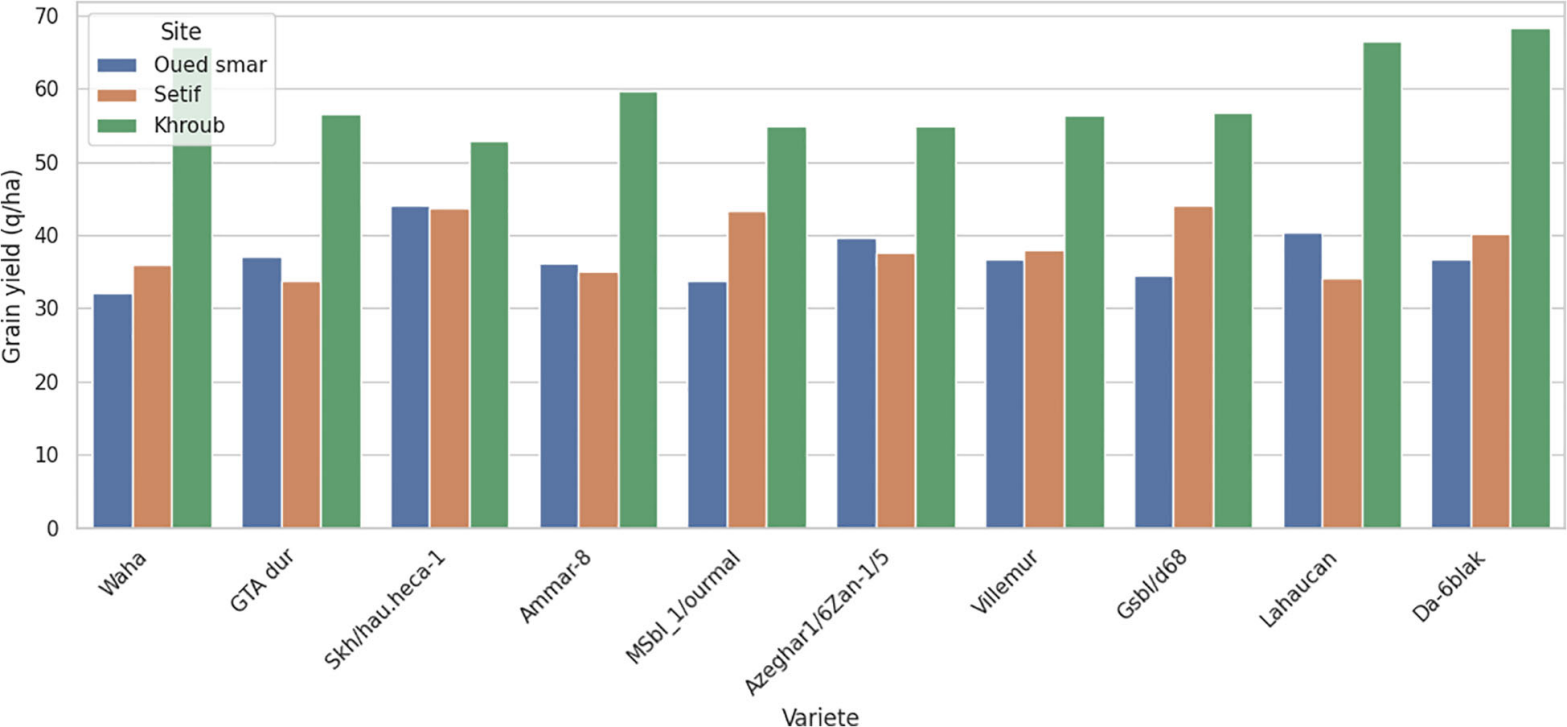
Grain yield (t ha^−1^) of *Durum wheat* genotypes across three experimental sites.

Significant differences in grain yield were observed among the three experimental sites over the five growing seasons (2021–2025) (**Figure 2**). Oued Smar showed the lowest average yield levels, Sétif showed intermediate performance, and El Khroub recorded the highest grain yields.

ANOVA revealed highly significant effects of environment (*p <* 0.001), genotype (*p <* 0.001), and their interaction (G×E, *p <* 0.001) on grain yield. These results indicate marked differences in yield performance across sites and genotypes.

The average yields over the five-year trial period showed a variation between regions exceeding 50%, ranging from 32 q ha^−1^ (Oued Smar) to nearly 68 q ha^−1^ (Khroub). Similarly, the Waha genotype exhibited the highest yield in Khroub (approximately 65 q ha^−1^), whereas the yield in Oued Smar was generally the lowest for all genotypes. Skh/hau.heca-1, Ammar-8, and Lahaucan also recorded high yields at El Khroub. The Sétif genotype showed intermediate performance for most genotypes.

Across environments, Waha and Ammar-8 maintained relatively high yield levels compared with the other genotypes, although genotype ranking varied according to site.

### 3.2 Phenotypic plasticity and genotypic stability along a water stress gradient

**Figure 3:**
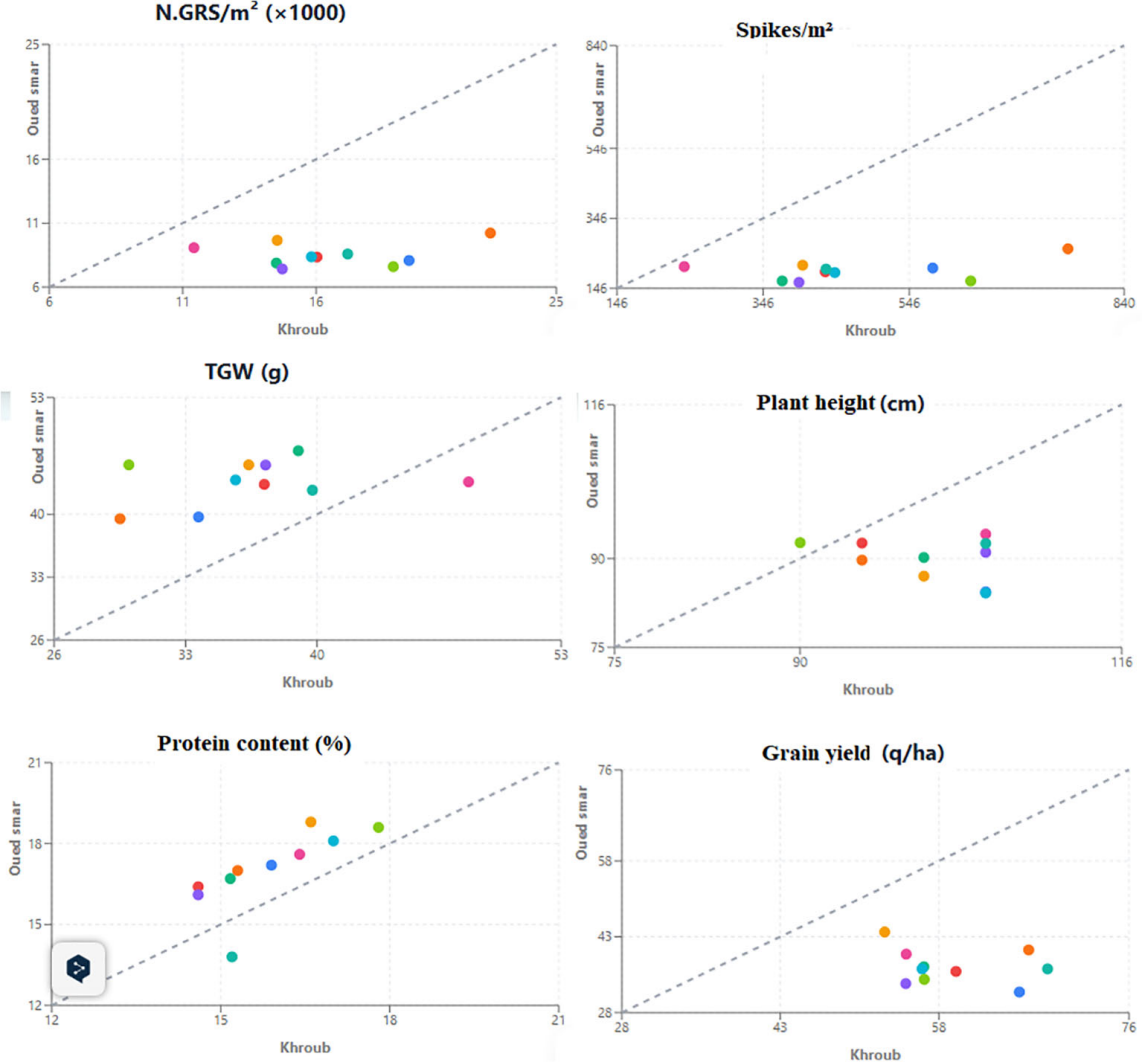
Comparison of agronomic performance between the Oued Smar and Khroub varieties for different wheat traits.

**Figure 4:**
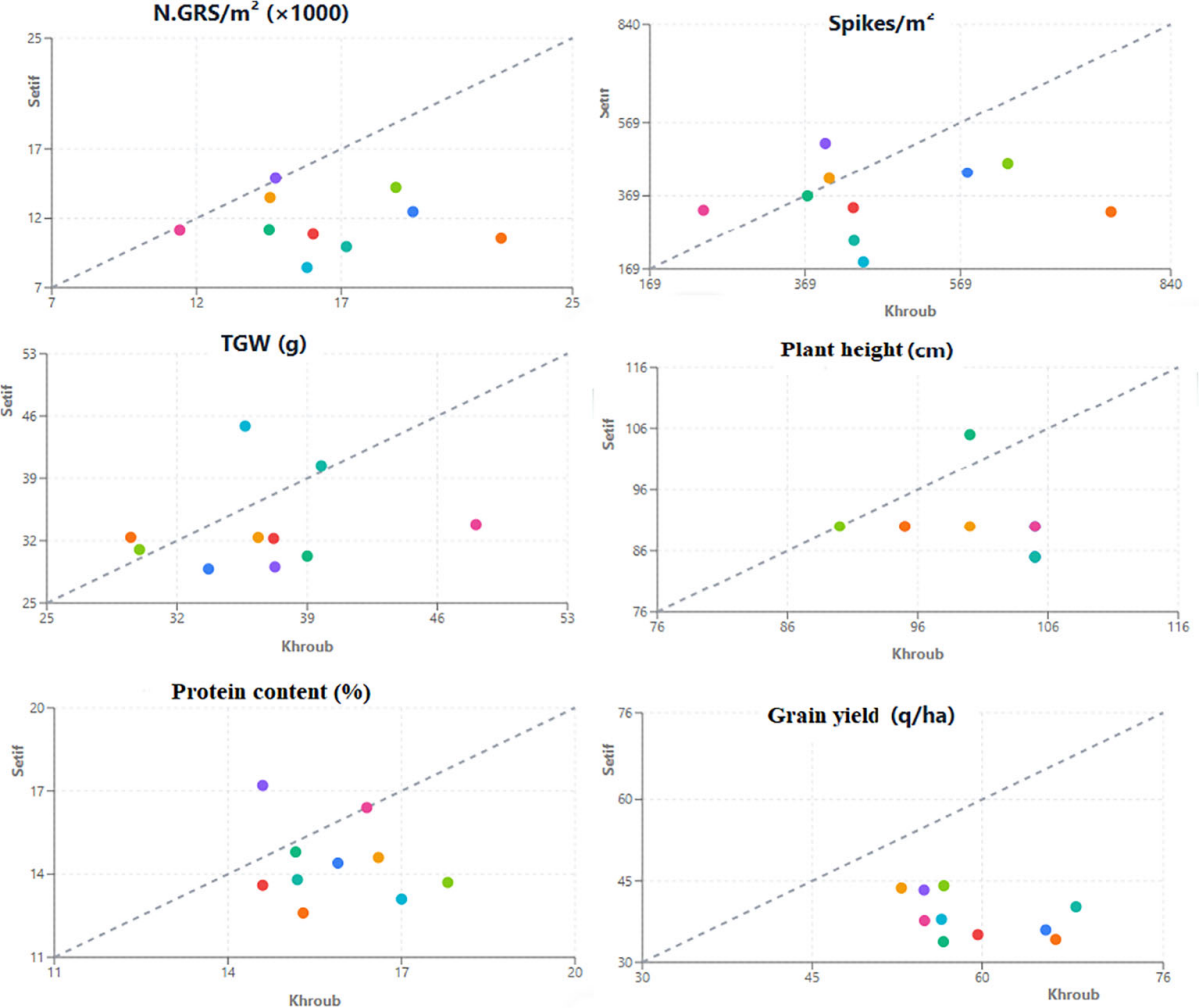
Correlations between the Sétif and Khroub varieties for wheat agronomic characteristics.

**Figure 5:**
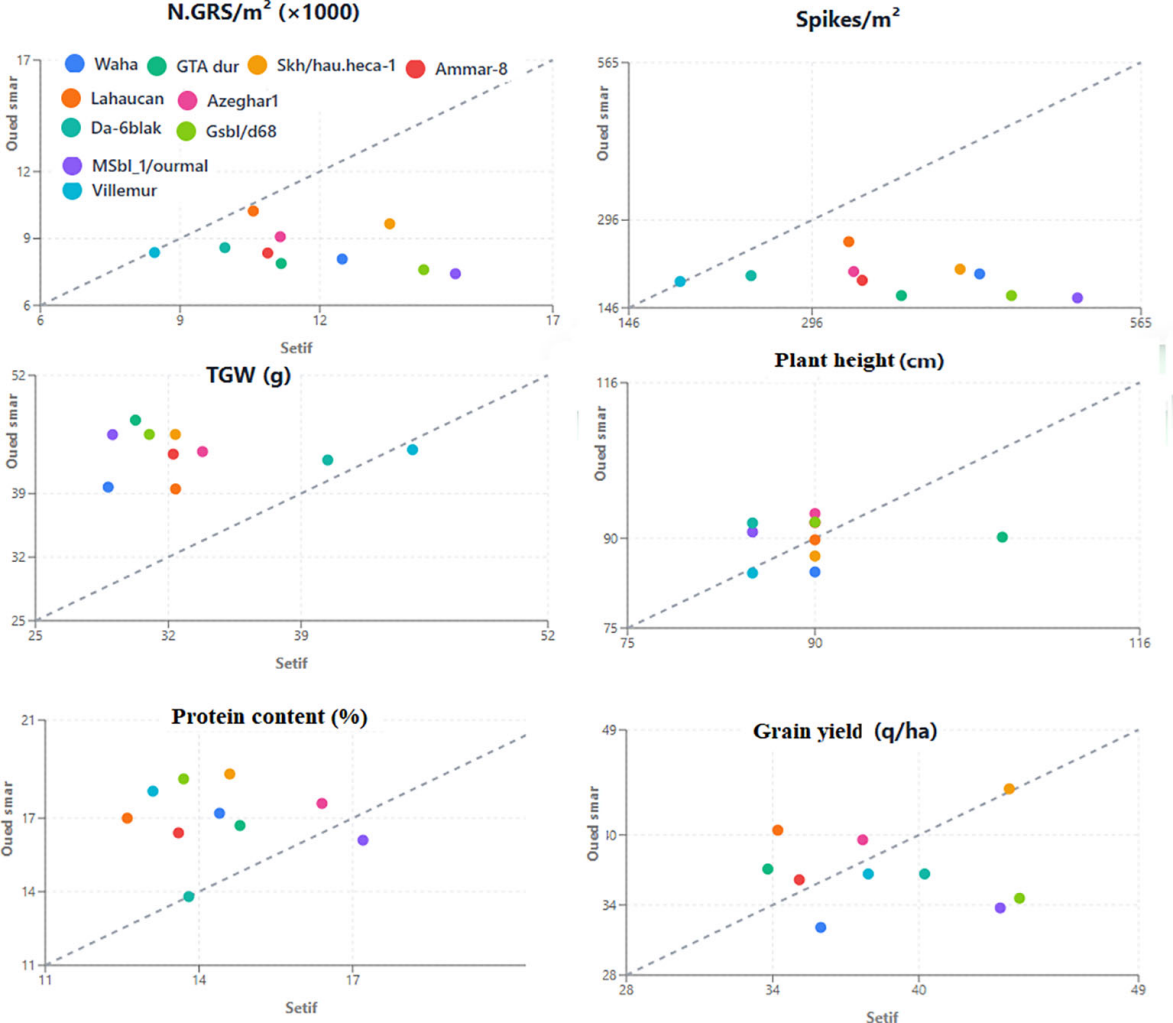
Comparative performance of ten wheat varieties across the Oued Smar and Sétif environments.

As shown in **Figures 3**, **4** and **5**, cumulative water deficit differed among the three sites, with values of 150mm at Khroub (E3), 380mm at Sétif (E2), and 560mm at Oued Smar (E1). The response of six durum wheat traits — grain yield, number of grains per square meter (N.GRS m^−2^), thousand-grain weight (TGW), number of ears per square meter, plant height, and protein content — varied across this gradient. Pairwise comparisons between sites showed deviations from the 1:1 line, indicating differences in genotype performance among environments.

High plasticity was observed for N.GRS m^−2^, with a marked reduction of up to approximately 35% at Oued Smar and Sétif (**Figure 3**), and maximum values observed at Khroub. Similarly, a 28–30% decrease in the number of ears per square meter (from approximately 450 to 320 m^−2^) was observed across the sites.

The thousand-grain weight (TGW) showed relative stability across environments (**Figure 4**), with a slight decline of approximately 10%, and a distribution of points close to the stability line (1:1 ratio). Plant height decreased by approximately 25% (from 85 to 65cm), indicating moderate plasticity across environments.

A 20% increase (from 11 to 13.5%) in grain protein content was observed under severe stress (**Figure 5**). In parallel, grain yield decreased sharply, reaching nearly 50% (from 32 to 16 q ha^−1^).

At the genotype level, Waha showed limited variation across the evaluated traits, whereas Ammar-8 expressed high yield under favorable conditions in Khroub (up to 2.8t ha^−1^, YSI = 0.89; (Ammar et al., 2004)). In contrast, yield reductions exceeding 50% were observed for GTA dur and Azeghar1/6Zan-1/5 under stress conditions.

A significant G×E crossover effect was observed between the performances in Sétif and Khroub (**Figure 4**). The ranking of genotypes varied according to the environment. The three pairwise comparisons (**Figures 3**, **4** and **5**) showed that the contrast between sites was associated with differences in the magnitude of G×E interaction, with the lowest correlation observed between Oued Smar and Khroub (**Figure 3**, *r* = 0.42) and the highest between Sétif and Khroub (**Figure 4**, *r* = 0.58).

### 3.3 Genotype stability and G*×*E interaction based on ASI and AMMI analysis

**Figure 6:**
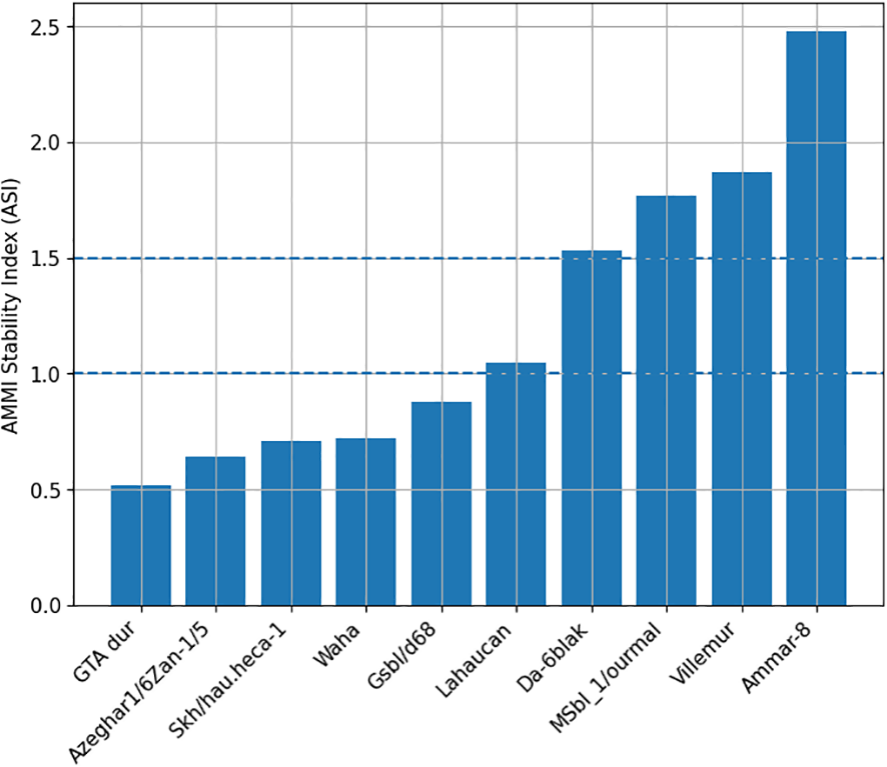
Genotypic stability according to the ASI, AMMI Stability Index for ten *Durum wheat* genotypes evaluated across three environments in Algeria. Higher stability is indicated by lower ASI scores. The horizontal lines show the stability categorization threshold values (ASI *<* 1.0: extremely stable; 1.0–1.5: moderately stable; *>* 1.5: unstable).

Among the 10 genotypes assessed, Waha (*ASI* = 0.72) and Skh/hau.heca-1 (*ASI* = 0.71) had the lowest ASI values, as shown in **Figure 6**. These genotypes also maintained relatively high average grain yields (44.56 and 46.84 q ha^−1^, respectively).

In contrast, Ammar-8 (*ASI* = 2.48) and Villemur (*ASI* = 1.87) showed the highest ASI values, together with marked differences in productivity between Khroub and Oued Smar.

PC1 and PC2 explained 73.8% and 18.4% of the G×E interaction variance, respectively, giving a cumulative value of 92.2% (**Table 4**), whereas the values presented in **Table 4** correspond to genotype scores on these two axes.

**Table 4:** Principal component scores for ten *Durum wheat* genotypes evaluated across three environments in Algeria.

Genotype	ASI	PC1	PC2
GTA dur	0.52	−0.24	+0.18
Azeghar1/6Zan-1/5	0.64	−0.28	+0.31
Skh/hau.heca-1	0.71	+0.28	−0.22
Waha	0.72	+0.32	+0.19
Gsbl/d68	0.88	+0.67	−0.45
Lahaucan	1.05	+0.89	+0.52
Da-6blak	1.53	+2.31	+0.87
MSbl_1/ourmal	1.77	−1.58	+1.12
Villemur	1.87	−1.47	−1.34
Ammar-8	2.48	+1.42	+1.89

*PC1* and *PC2*, Principal component scores from AMMI analysis.

Site-to-site correlations showed a stronger association between Khroub and Sétif (*r* = 0.58) than between Khroub and Oued Smar (*r* = 0.42).

#### 3.3.1 Inter-site correlations supporting AMMI-based G*×*E interpretation

**Figure 7:**
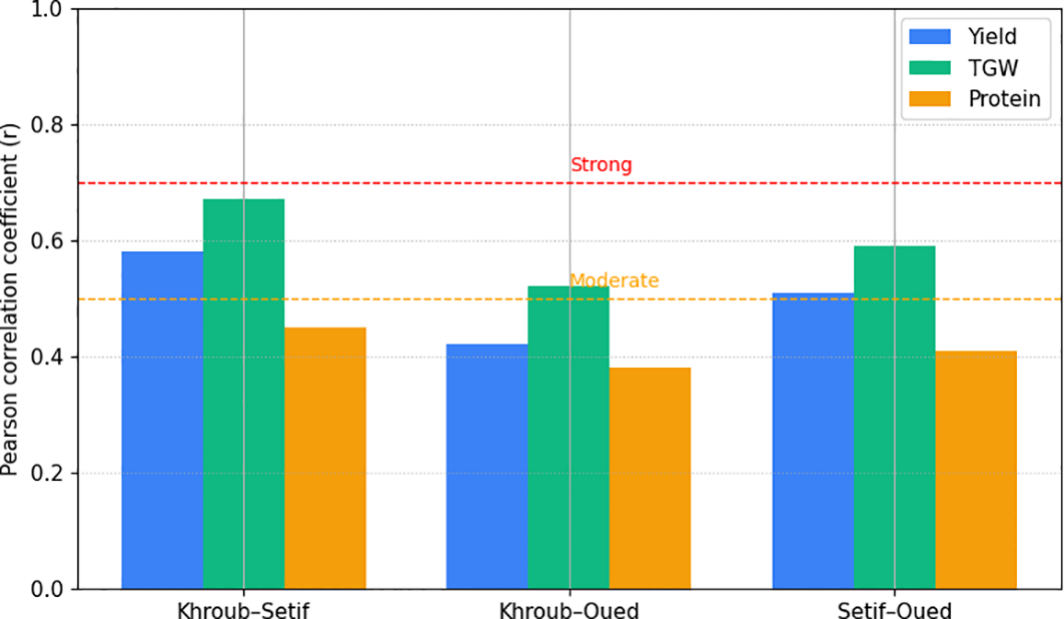
Inter-site Pearson correlations for yield and quality-related traits.

The Pearson correlations between the experimental sites of Khroub, Sétif, and Oued Smar for yield, TGW, and protein content are shown in **Figure 7**. The results showed that TGW had the highest correlations between sites, with values estimated to be quite strong (*r* = 0.52–0.67). Yield correlations were lower, particularly between Khroub and Oued Smar (*r* = 0.42), whereas the correlation between Khroub and Sétif reached *r* = 0.58.

### 3.4 SHAP-based interpretability of machine learning predictions for yield drivers

**Figure 8:**
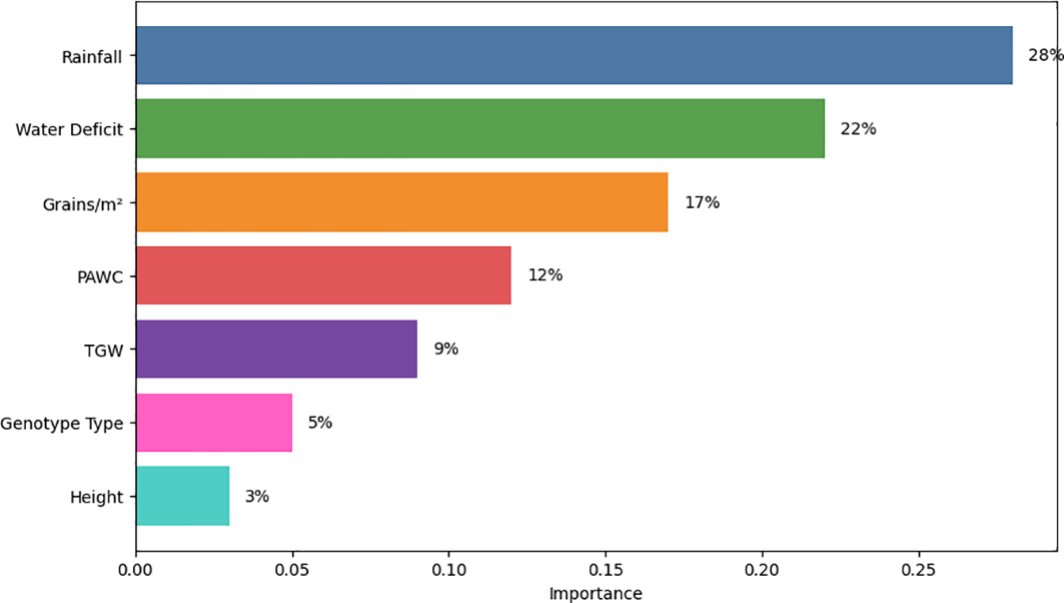
SHAP summary plot showing the relative importance of environmental, agronomic, and genotypic variables in predicting *Durum wheat* grain yield. Variables are ranked by their mean absolute SHAP values, with precipitation (Precipitation), water deficit (Water Deficit), and grain number per square meter (N.GRS/m^2^) being the most influential predictors.

The relative importance of environmental, agronomic, and genotypic variables in predicting grain yield, as derived from the SHAP analysis of the best-performing machine learning model, is illustrated in **Figure 8**. Precipitation (28%) and water deficit (22%) were the dominant predictors, together accounting for 50% of the model’s total explanatory power. Grain number per m^2^ (18%) and soil plant-available water capacity (12%) ranked third and fourth, respectively, followed by thousand-grain weight (9%), genotype type (5%), and plant height (3%).

### 3.5 Synthesis: multi-criteria genotype classification for targeted deployment

The combination of AMMI stability indices, drought tolerance indices (STI, DSI, and YSI), and machine learned variable importance supported a multi-criteria classification of the ten *Durum wheat* genotypes into four categories of adaptation (**Table 4**).

The Waha and Skh/hau.heca-1 genotypes were characterized by low ASI values (0.72 and 0.71, respectively), high YSI values (0.89 and 0.87, respectively), and moderately high yields across environments.

Ammar-8, Da-6blak, and Lahaucan recorded high grain yields at Khroub (59.62–68.29 q ha^−1^) but showed lower performance at Oued Smar, with yield decreases of 32–46%. These genotypes also had relatively high ASI values (1.05–2.48) and YSI values ranging from 0.61 to 0.74.

GTA dur and Azeghar1/6Zan-1/5 showed YSI values between 0.78 and 0.82, together with intermediate ASI values (0.52–0.64), whereas Villemur, MSbl_1/ourmal, and Gsbl/d68 showed lower YSI values (0.55–0.69) and higher instability across environments.

## 4 Discussion

### 4.1 Environmental drivers of genotypic performance under Mediterranean rainfed conditions

The strong yield gradient observed across the three sites, together with the highly significant effects of environment, genotype, and G×E interaction on grain yield, confirms that single-environment selection is insufficient for identifying broadly adapted genotypes under Mediterranean rainfed conditions (Ceccarelli, 1989; Mohammadi et al., 2022).

The progressive nature of water stress and the increasing frequency of heat waves linked to climate change in the Mediterranean area accentuate the need to select genotypes with adaptive characteristics for water shortages and high temperatures. Climate projections for Algeria indicate rising temperatures during the wheat growth cycle, reduced rainfall, and increased drought frequency, which are major constraints on wheat cultivation in this region.

The differences in yield observed among Oued Smar, Setif,´ and El Khroub suggest that local environmental conditions, including water availability and soil properties, played a major role in determining genotype performance. The superior yields recorded at El Khroub indicate that this site provided more favorable conditions for grain production, whereas the lower yields observed at Oued Smar reflected stronger environmental limitation.

Likewise, the relatively stable performance of Waha and Ammar-8 across environments suggests useful adaptive potential under contrasting Mediterranean conditions. The superior performance of Waha under both favorable and limiting conditions supports its value as a stable genotype, while the strong response of Ammar-8 under favorable conditions indicates a higher degree of environmental sensitivity.

Regardless of their breeding background, the superior performance of the Algerian landrace Waha and the ICARDA-improved cultivar Ammar-8 demonstrates their good tolerance to restrictive environmental conditions. This yield stability, which reflects increased heat tolerance and phenological plasticity in response to climatic stress, makes both genotypes suitable candidates for varietal improvement programs aimed at enhancing durum wheat resilience in Mediterranean rainfed environments.

### 4.2 Phenotypic plasticity of yield components along the water stress gradient

In contrast to the relative stability of TGW, the marked plasticity of N.GRS m^−2^ and ear density across the water stress gradient indicates that these traits were more responsive to environmental variation, which is consistent with the recognized hierarchy of yield component sensitivity in water-limited environments (Slafer et al., 2014).

N.GRS m^−2^ is the main determining factor of yield under water stress and is particularly sensitive to climatic constraints during stem elongation and flowering. Similarly, the reduction in ear density reflects a decline in tillering under increasing stress.

The decline in N.GRS m^−2^ and ear density across the stress gradient suggests that grain number was one of the main drivers of yield reduction under water-limited conditions. By contrast, the relative stability of TGW indicates stronger genetic buffering and possible compensatory effects when grain number declines ()?.

The relative stability of TGW across environments can be explained by the importance of genetic control and partial compensation when the number of grains is reduced. The compensatory processes of grain replenishment involved in protecting individual grain production when source constraints decrease sink demand are linked with this relative insensitivity of TGW to moderate stress (a decrement of 10% versus 35% for the number of grains ())?. However, similar compensatory effects break down under terminal stress, as observed at Oued Smar, leading to cascading yield reductions when both components decline simultaneously.

The increase in grain protein content under severe stress is consistent with a concentration effect caused by reduced grain yield, a pattern commonly reported in Mediterranean environments. This inverse relationship between yield and protein concentration supports the interpretation that stress modifies both productivity and grain quality.

The 20% increase in grain protein content under severe stress is explained by the concentration effect linked to yield reduction, which is an inverse relationship typically observed in Mediterranean environments (López-Bellido et al., 1998; De Santis et al., 2021). This accumulation of proteins caused by stress frequently results in a passive concentration effect: a decrease in grain production increases the relative percentage of proteins without a corresponding increase in the absolute protein yield per hectare. Moreover, protein quality often deteriorates under stress, with gluten composition shifting toward higher gliadin:glutenin ratios and weakened dough rheology (Li et al., 2013; Zahra et al., 2023), which jeopardizes pasta quality despite apparently higher protein content.

### 4.3 Genotype stability, G*×*E interaction structure, and the yield–stability dilemma

Simple comparisons of means or univariate analyses do not allow for the disaggregation of the main effects (genotype and environment) or the identification of the structure of the interaction. An AMMI-type analysis for interaction decomposition allows the combination of analysis of variance (ANOVA) for the decomposition of additive effects (G and E), followed by principal component analysis (PCA) to identify G×E interactions.

The AMMI-type analysis used here combines ANOVA for the decomposition of additive effects (G and E) with principal component analysis (PCA) to characterize G×E interactions (Gauch, 2006; Yan et al., 2000). The first two principal components explained 92.2% of the G×E interaction (PC1: 73.8%, PC2: 18.4%), and the two-factor model suggests that Khroub and Sétif constitute a distinct mega-environment, separate from Oued Smar, consistent with the inter-site correlations (*r* = 0.58 for Khroub–Sétif vs. *r* = 0.42 for Khroub–Oued Smar) (Senoussi et al., 2020).

The 4.8-fold difference observed in the present study between the most stable genotype (GTA dur: *ASI* = 0.52) and the least stable (Ammar-8: *ASI* = 2.48) is consistent with ranges reported in comparable studies. Tahir et al. (2023) found that ASV values varied from 4.7 for the most stable genotypes to 37.7 for the least stable in a study carried out in 24 heat-stressed environments in Sudan, confirming that Mediterranean and Sub-Saharan rainfed systems share comparable patterns of genotypic instability under thermal and water stress. Furthermore, the proportion of G×E interaction explained by the first two AMMI components (92.2%; PC1: 73.8%, PC2: 18.4%) is comparable to results reported for similar Mediterranean environments. In central Greece, the GGE biplot evaluation of 15 *Durum wheat* varieties across six environments explained 73.06% of the overall variability and distinguished two distinct mega-environments associated with low- and high-productivity systems (Kyratzis et al., 2023), a pattern consistent with the mega-environment structure identified in the present study between El Khroub–Sétif and Oued Smar.

A curious paradox emerges from this analysis: genotypes achieving maximum yield potential under favorable conditions (Type II: Da-6blak 68.3 q ha^−1^, Lahaucan 66.5 q ha^−1^ at Khroub) suffered disproportionate yield losses under stress (reductions of 46% and 39% at Oued Smar) and showed high instability (ASI = 1.53 and 1.05, respectively). Conversely, broadly adapted Type I genotypes (Waha *ASI* = 0.72, Skh/hau.heca-1 *ASI* = 0.71) maintained stability at the expense of maximum yield. The yield–stability trade-off can potentially be resolved through the introgression of stability alleles from locally adapted landraces into high-yielding elite lines (Royo et al., 2007; Nachit and Elouafi, 2004).

These results are consistent with findings reported for Mediterranean *Durum wheat*. In a study of eight genotypes evaluated across 15 environments in Jordan, modern improved cultivars were found to be consistently more stable than traditional local varieties (Nachit and Elouafi, 2004), which may explain the superior stability of the more recently selected genotypes in the present study. Furthermore, an examination of 12 stability criteria for Mediterranean *Durum wheat* revealed that AMMI-derived indices (ASV and AWAI) consistently clustered and showed weak correlations with regression-based stability measures, thus confirming the complementarity of ASI and Finlay–Wilkinson parameters used in the present study.

The multi-criteria classification also highlights contrasting patterns of adaptation among genotype groups. Waha and Skh/hau.heca-1 combined low ASI values with relatively high yields across environments, supporting their classification as broadly adapted and stable genotypes. In contrast, Ammar-8, Da-6blak, and Lahaucan expressed higher yield potential under favorable conditions but stronger yield penalties under stress, indicating specific rather than broad adaptation. GTA dur and Azeghar1/6Zan-1/5 showed more moderate but relatively consistent responses, whereas Villemur, MSbl 1/ourmal, and Gsbl/d68 appeared less stable and less adapted to the tested environments.

### 4.4 Machine learning interpretability: bridging MET analysis and physiological yield drivers

A significant methodological contribution of this study is the integration of classical multi-environment trial (MET) analysis with machine learning interpretability tools. Conventional AMMI and GGE analyses operate in linear or multiplicative frameworks that may overlook the nonlinear dynamics of stress response (Malosetti et al., 2013), whereas SHAP-based variable importance provides a complementary, physiologically interpretable layer of analysis.

Precipitation (28%) and water deficit (22%) dominated yield prediction, together accounting for half of the model’s total relevance (**Figure 8**).

This percentage is strikingly similar to that of recent meta-analyses carried out in Mediterranean settings, where 55–65% of the variation in cereal yield was explained by water-related factors (Araus et al., 2002), supporting the idea that water scarcity remains the primary barrier to productivity in semi-arid rainfed systems ()?. The third-ranked predictor, grain number per m^2^ (18%) confirms its established position as the yield component most vulnerable to environmental perturbations (Slafer et al., 2014). The 12% contribution of soil plant-available water capacity (PAWC) reflects the interaction between root depth and soil physical properties, underscoring the importance of the genotype–soil nexus in rainfed systems (Sadras and Lawson, 2011; Kirkegaard and Hunt, 2010).

### 4.5 Mechanistic basis of adaptation and recommendations for targeted genotype deployment

The multi-criteria classification of genotypes into four adaptation types, combining ASI, drought tolerance indices (STI, DSI, YSI), and machine-learned variable importance, provides a scientifically grounded framework for variety deployment in the rainfed cereal-growing regions of Algeria. The higher stability of Type I genotypes (Waha, Skh/hau.heca-1) is likely due to the coordinated expression of hardiness adaptation characteristics, acting from the molecular level (osmotic adjustment, antioxidant systems) to the whole-plant level (root architecture, phenology) (Cattivelli et al., 2008; Blum, 2011).

Tolerant varieties maintain photosynthetic capacity through proline and soluble sugar accumulation, sustained chlorophyll biosynthesis, and stimulated antioxidant enzymatic activity, thereby preventing oxidative damage and allowing continuous assimilation for grain filling even under stomatal closure (Ltaief and Krouma, 2023; Blum, 2017). Root architecture is an additional critical factor: drought-tolerant genotypes exhibit 30–50% higher root biomass allocation and deeper root systems (over 120cm versus 80cm in sensitive genotypes), enabling the uptake of residual soil moisture from deep horizons (MansChadi et al., 2006; Li et al., 2023; Rossi et al., 2024).

From an agronomic perspective, these patterns support the preferential use of stable genotypes such as Waha in marginal rainfed environments, while higher-yielding but more environment-sensitive genotypes may be better suited to favorable or managed production conditions.

Type II genotypes (Ammar-8, Da-6blak, Lahaucan) are recommended for intensive production systems in favorable environments such as Khroub, where water constraints can be mitigated. Type III genotypes (GTA dur, Azeghar1/6Zan) are suited to moderately stressed environments such as Sétif, where their drought tolerance confers a competitive advantage. Type IV genotypes (Villemur, MSbl_1/ourmal, Gsbl/d68), despite their poor direct performance, represent valuable genetic diversity for introgression into breeding programs. The integration of selection for vernalization and photoperiod genes (VRN and PPD) offers a further avenue for adjusting phenology to local environmental conditions, as increasingly implemented by CIMMYT and ICARDA breeding programs (Crespo-Herrera et al., 2021; Zheng et al., 2013).

### 4.6 Yield-quality trade-offs under mediterranean drought: implications for end-use functionality

The deep impact of hydric stress on the technological characteristics of grains, which determine the functionality of the end product, is sometimes overlooked in the adaptation to harshness. Our findings of a 20–32% increase in protein concentration under stressful conditions superficially suggest an improvement in nutritional quality and processing quality. However, recent studies published in the journal Foods show that this accumulation of proteins caused by stress frequently results in a passive concentration effect: a decrease in grain production increases the relative percentage of proteins without a corresponding increase in the absolute yield of proteins per hectare (López-Bellido et al., 1998; De Santis et al., 2021). Moreover, protein quality, distinct from quantity, often deteriorates under stress, with gluten composition shifting toward higher gliadin:glutenin ratios, reduced high-molecular-weight glutenin subunit (HMW-GS) polymerization, and weakened dough rheology (Li et al., 2013; Zahra et al., 2023).

Specifically, stress-induced disruptions in nitrogen metabolism during grain filling alter the timing of storage protein synthesis, leading to the asynchronous accumulation of gliadin monomers (which confer dough extensibility) relative to glutenin polymers (which provide elasticity and strength) (Zib et al., 2025; Wang et al., 2024). According to Dexter and Marchylo (2000) and Sissons (2022), a high-performing semolina requires a dense glutenin/gliadin ratio and a high concentration of high-molecular-weight glutenin (HMW-GS) to withstand high temperatures without undergoing structural breakdown. Although the protein content is higher than that of Oued Smar (17.1% on average), there are likely underlying compositional imbalances that jeopardize the quality of pasta production. The next steps in this study should include a direct assessment of gluten force indices (Zeleny sedimentation, SDS sedimentation, and mixograph parameters) and profiling of prototypical subunits to fully characterize the evolution of quality under stressful conditions (Kartseva et al., 2023, 2024).

### 4.7 Emergence of eco-environments and development of a stabilization-focused adaptation

With respect to adaptation, we can show that the present-day categorizations of marginal environments, like Oued Smar, are distinct meta-environments and have low correlation with more desirable locations. Stability and climate-smart agricultural activities are not optional but essential since such stressful environments are vulnerable to spatial and temporal changes with respect to projected climate change. In turn, the synchronized approach incorporating the use of resilient cultivars, water- and soil-conservation methods, proper nutrient management, and flexible sowing plans with institutional structures that enable quick variety introduction and active farmer participation is a prerequisite for efficient adaptation.

## 5 Conclusions

Agronomic and genotypic characteristics are less important as yield drivers than environmental water related variables, according to SHAP-based interpretation of the best-performing machine learning model. Grain number per m^2^ (N.GRS m^−2^) emerged as the most significant agronomic predictor, consistent with its well-established sensitivity to water stress during stem elongation and flowering.

The multi-criteria classification system offered a scientifically grounded framework for targeted varietal deployment. Broadly adapted and stable genotypes (Type I) are recommended for all rainfed environments, including marginal zones. High-yielding but environmentally sensitive genotypes (Type II) are better suited to intensive and favorable production systems. Moderately stable and drought-tolerant genotypes (Type III) perform best under moderate water stress. Poorly adapted genotypes (Type IV), while unsuitable for direct deployment, remain valuable genetic resources for introgression breeding programs.

Finally, the considerable environmental dependence of grain quality parameters — particularly protein content, wet gluten, and the Zeleny sedimentation index — and the stress-induced passive protein concentration effect highlight a yield–quality trade-off that must be explicitly accounted for in multi-environment breeding programs, beyond yield stability criteria alone.

Overall, the combination of explainable machine learning with classical multi-environment trial analyses provides an operationally scalable decision support framework for climate-resilient *Durum wheat* breeding in Mediterranean semi-arid regions facing increasing climate uncertainty.

## Data Availability

The raw data supporting the conclusions of this article will be made available by the authors, without undue reservation.
